# A study on the effectiveness of narrative image types, message framing, and psychological distance in enhancing young people's self-efficacy in marine garbage recycling

**DOI:** 10.1016/j.heliyon.2024.e34919

**Published:** 2024-07-21

**Authors:** Mo Chen, Kexin Chen, Yang Qin, Yanfei Zhu

**Affiliations:** a College of Art and Design, Nanjing Tech University, Nanjing, China; b School of Mechanical Engineering, Southeast University, Nanjing, China

**Keywords:** Garbage recycling, Cartoon, Message framing, Psychological distance

## Abstract

Intentional or unintentional littering, especially among young people, poses a threat to the marine environment. It is, therefore, necessary to enhance awareness of marine environmental protection among youth groups. This study explored the interaction between visual image types (photograph vs. cartoon), message framing (positive vs. negative), and psychological distance (human vs. turtle). A three-way analysis of variance (ANOVA) and regression analysis were used to verify the hypotheses. The key findings of the research were as follows: (1) Among the eight information construction methods, cartoons combined with turtles and presented with positive outcomes were the best way to communicate, i.e., cartoons showing animals in a friendly and positive way were the most effective form of marine garbage recycling for young people, and (2) Young people's preference for information will promote their self-efficacy in marine garbage recycling. The implications of these findings for developing marine protection information communication education for youth groups are also proposed.

## 1 Introduction

Marine litter, the majority of which is plastic, has caused serious damage to the marine environment [1]. Plastics, such as food wrappers, plastic bottles, caps, and bags have severely impacted marine biodiversity [2]. In addition, marine debris affects tourism [[3], [4], [5]], food safety [6], human health [7], navigation, and fishing [1]. Studies have shown that tourism is a major source of marine debris, especially on small islands where tourism-related activities can generate twice the marine debris compared to local waste production[3^,^5^,^8]. To mitigate this issue, coastal areas have implemented various measures to encourage tourists to recycle, such as reward and punishment systems, moral appeal and education, increased patrolling of scenic spots, etc. [3]. However, the effectiveness of these measures has been minimal.

Research indicates a significant correlation between marine littering and tourism [4], suggesting that the rapid growth of tourism has led to an increase in marine plastic litter, largely due to tourists intentionally or unintentionally littering [8]. Surveys in coastal industrial cities have found that younger people (18–36 years old) are less likely to recycle compared to older tourists [8]. Further studies on youth not recycling identified laziness, indifference, and a lack of awareness of the problem and its consequences as the main reasons for their behavior [9]. The attitudes and behaviors of young tourists in tourism activities are crucial for sustainable tourism development. Given that the younger generation is responsible for protecting the current and future marine environment, it is essential to raise awareness of the marine litter problem among young tourists and to enhance their sense of Self-Efficacy (SE) in recycling marine litter [10]. As Albert Bandura stated, “behavior greatly depends on perceived self-efficacy”, which refers to “beliefs in one's capabilities to mobilize the motivation, cognitive resources, and courses of action needed to meet given situation [[11], [12], [13]].” In social cognitive theory, self-efficacy is a critical behavioral change component. Information communication is an effective means of awareness training [14]. Effective information communication can get the support of the public to improve their knowledge, attitudes, and behaviors, which in turn can enhance their marine literacy [10].

The framing effect, a cognitive bias where differences in information presentation can influence decision-making, is effective in shaping young people's behaviors and motivating them to change [15]. Joachim Claudet and other scientists, ecologists, and ocean advocates assert that educational information communication can change social norms and behaviors, which is crucial for achieving the United Nations' goals outlined in the “Decade of Marine Science for Sustainable Development” (2021–2030) [16]. To cultivate awareness of the marine litter problem among the younger generation and establish effective methods for disseminating marine conservation information, much research has focused on school-age children, yielding positive results. However, the limited mobility of schoolchildren highlights the necessity of involving a broader audience [6]. In retrospect, young people have played an important role in civic engagement. They possess higher levels of education, the ability to access information independently, and view marine conservation as an important value. This study aims to provide ongoing educational support on the topic of marine litter, seeking to develop engaging and educational marine conservation communication through various narrative image types (photos and cartoons), combined with message framing and psychological distance. The goal is to enhance awareness and self-efficacy among youth regarding the issue of marine plastic pollution.

## 2 Related works

### 2.1 Effectiveness of photos and cartoons in environmental protection information communication

With the advent of the Internet era, information communication has become more convenient and faster than ever before. Young people now have access to media in increasingly diverse formats. Since more than 80 % of human information is obtained visually, the value of visual narratives has been recognized early on. To increase participation, and attention among young people and to influence them emotionally, visual narratives typically combine various media formats (text, pictures, and video) to create compelling stories [17]. Among these media, pictures have a unique ability to convey visual information, emphasizing the adage that “a picture is worth a thousand words.” This highlights the powerful effect of images in conveying information and attracting attention. Therefore, carefully selected and designed pictures play a crucial role in effective communication.

Photos and cartoons are different types of pictures frequently used in environmental information communication. Photographs are external, permanent, static images captured using a camera, representing real objects. Some studies claim that photographs are powerful because they can provoke individuals' memories and emotions. In pro-environment messages, photographs depicting natural scenery can affect viewers' behavioral intentions [18]. For example, a photo of a beach full of plastic waste can evoke disgust in the viewer, while a sea turtle deformed by plastic waste can evoke sadness. All of these photos, which evoke different emotions, have the effect of persuading consumers to reduce their plastic consumption [19]. Photos from marine protected areas are more positively viewed and more popular compared to those from non-protected areas [20]. Motivating photographs on travel websites has been found to significantly enhance viewers' willingness to travel to a destination [21]. Collectively, these claims demonstrate that photographs play an important role in shaping viewers’ attitudes and behavioral intentions [22].

In contrast, cartoons are less realistic than photographs. The word cartoon has several meanings and can refer to a comic or animation, but in this context, it is used to refer to drawings that are less realistic [23]. Cartoons are not only for entertainment but are also considered suitable for delivering serious ecological messages [23]. Some studies have shown that well-designed cartoons are more effective in conveying deep and complex information to a wide audience than most scientific articles [23]. Brown and Lindvall proposed that cartoons function as pedagogical tools providing exemplary or revelatory parables. They can promote public pro-environment beliefs and encourage appropriate behavior entertainingly. Some scholars suggest that cartoons are a friendly and positive way to persuade large public audiences to recognize the importance of conserving biodiversity, especially in the face of climate change, extinction, and habitat destruction. To develop environmental awareness among elementary school students, some people proposed that educational cartoons be incorporated into the formal environmental education curriculum. Cartoons can spark children's imagination and encourage them to act. This strategy is therefore considered an effective way to build sustainable environmental awareness for the next generation [24]. Studies have shown that representing coral reefs in cartoons can improve students' attitudes toward the coral reef ecosystem [25]. In summary, cartoons are regarded as an effective way to educate students about the environment and curb environmental problems.

From the above, it can be observed that photographs present environmental issues in a realistic and objective manner to emphasize the authenticity and urgency of these problems. In contrast, cartoons establish an emotional connection and simplify content to resonate with viewers, thereby attracting a broader audience and motivating them to take environmental action. Both media have a unique value in environmental information communication. As previously mentioned, communication tools should combine with other aspects of information construction to improve the effectiveness of communication [26]. This study will explore the most appropriate and effective communication tools for engaging youth groups.

### 2.2 Message framing and psychological distance in marine garbage recycling

Message framing is critical in environmental information communication. It emphasizes either the positive effects (e.g., environmental improvement, cost savings) of solving environmental problems or the negative effects (e.g., environmental destruction, financial losses) of failing to address them. Over the past few decades, the effectiveness of positive versus negative framing has been extensively studied. It is generally believed that negative-framed messages are more effective in changing harmful behaviors, especially in addressing contemporary issues like natural resource conservation [27], air pollution [28], etc.

The integrity of the landscape is crucial for the development of the tourism industry [4]. As coastal tourists leave behind increasing amounts of waste, the rural scenery gradually deteriorates, leading to a decline in tourism [29]. A study using Phu Quoc Island in Vietnam as a case example found that tourism has led to increasing plastic waste pollution, tarnishing the island's natural beauty and resulting in negative portrayals in Vietnamese media and international blogs [4]. Thus, marine debris, prominently represented by marine plastics, not only destroys the natural beauty of beaches and threatens the tourism economy, but also endangers marine life. To encourage tourists to recycle their garbage, it is essential to increase awareness among youth about the root causes of marine littering. This study argues that indiscriminate litter damages the marine environment and endangers marine life. Consequently, negative-framed messages should be more effective than positive-framed messages in persuading people not to litter. However, since most young people engage in tourism for pleasure and seek positive experiences, negative message framing may not be appropriate in this context. Some studies have shown that positively framed messages are more effective in tourism. For example, gain-framed messaging can increase tourists' willingness to pay for carbon offsetting. Positively framed biodiversity conservation messages (focused on gains and love) were more effective than negatively framed ones (focused on loss and warning), encouraging more public donations and support for conservation [30]. However, O'Keefe and Jensen noted that the difference between positive and negative frames was inconsistent and not always significant [31]. Thus, the effectiveness of message framing should be considered in conjunction with other factors.

Research also indicates that the degree of psychological distance influences the effectiveness of framing [32]. There are four dimensions of psychological distance: spatial (here vs. far away), temporal (now vs. future/past), social (me vs. others), and hypothetical (certain vs. uncertain) [33]. Psychological distance affects evaluations and behavioral intentions [33]. Hartley et al. found that the impact of messages on public awareness is enhanced when marine litter is represented as a current threat rather than a future one. Similar results have been found in other environmental communication contexts; for instance, climate change messages are more effective in encouraging action when localized [34]. Because psychological distance is egocentric, anchored at the self, here, and now, objects at the proximal end may have more influence than those at the other end of the spectrum [35]. Additionally, wildlife, compared to human activity, is passive and directly threatened by environmental damage [36]. Issues like not recycling are considered more harmful to nature than to humans [2]. Focusing on wildlife can be a powerful motivator for pro-environmental behavior. For example, polar bears and fish have been used to promote energy conservation, with the idea that inappropriate behavior could push these animals toward extinction [37]. Other examples include “Bambi” advocating against forest destruction, “Smokey Bear” defending forest ecology, and “Sea turtles” calling for the reduction of plastic waste. Emotional bonds with these animals can significantly strengthen viewers’ enthusiasm for nature protection.

### 2.3 The effect of information construction on viewers’ preference and self-efficacy

In everyday life, preferences are a way to express our attitudes, and attitude change and persuasion are core concepts in the study of individual behavior [38]. Persuasive messages are more effective when they match the preferences of the intended audience, provided the quality, credibility, and logic of the message are consistent [39]. Fang and Hsu emphasized that visual representations enhance the persuasive power of a message and users’ emotional reactions. Therefore, when factors such as information quality and credibility are consistent, aligning the message with audience preferences is crucial for successful persuasion.

Another important factor influencing attitudes and promoting behavior change is an individual's self-efficacy (SE), which refers to an individual's perceived competence to succeed at or accomplish a specific task. A literature review showed that individuals with higher SE have greater confidence in their ability to achieve goals, particularly in pro-environment contexts. Several studies confirm that higher SE can motivate environmental behavior and encourage engagement in more challenging pro-environmental actions [40,41]. Shafiei and Maleksaeidi found that for university students, environmental attitudes and SE directly determine pro-environmental behavior [42]. SE is also closely related to goal achievement [43], suggesting that higher SE can motivate individuals to protect the marine environment. Therefore, the effectiveness of communications is influenced by the types of images, message framing, and psychological distance. The impact on users' behavioral intentions is shaped by the interplay between these variables. Based on this, we propose the following hypotheses.

Hypothesis 1 Message framing and narrative image types will have an interactive effect on young people’s self-efficacy in marine garbage recycling. Specifically, positively framed messages presented as cartoons will enhance SE more than other combinations.

Hypothesis 2 There is an interaction effect between image types, message framing, and psychological distance on self-efficacy*.* Specifically, cartoons with positive framing and distal psychological distance (turtle) are the most effective in enhancing SE.

Hypothesis 3 Young people’s preference for information will enhance their self-efficacy in marine garbage recycling.

## 3 Method

### 3.1 Experiment design

In this study, we consider “Don't throw garbage” as an example of the impact on the marine environment. The independent variables are message framing (positive vs. negative), visual image types (photograph vs. cartoon), and psychological distance (human vs. turtle). The dependent variables are preferences and self-efficacy (SE) regarding the information construction. A 2 × 2 × 2 scenario-based experiment was conducted to analyze the effects of different types of information and their interactions. In addition, we investigated the relationship between viewer's preferences and SE using linear regression.

Marine plastic pollution exemplifies the negative impact of human activity on nature [19]. We illustrated its potential effects on humans and marine animals through eight visual stories. For narrative image types, we used hand-drawn stills from cartoon films, modified to create visual stories. Most of the photos were screenshots from documentary films. The presented information comprised similar images to help generate coherent and meaningful stories.

The story focusing on marine animals depicts turtles eating jellyfish, one of their main foods. For example, the positively framed scenarios (recycling) with distant psychological distance (i.e., turtle) showed the harmonious coexistence of sea animals (see Fig. 1) and abundant life (see Fig. 2). Conversely, negatively framed scenarios (drop litter at will) depicted adverse situations for sea turtles, such as fainting after eating plastic bags (in the cartoon, indicated by circles around the turtle's head) or being found dead on the beach (photograph; see Fig. 3, Fig. 4).

**Fig. 1:**
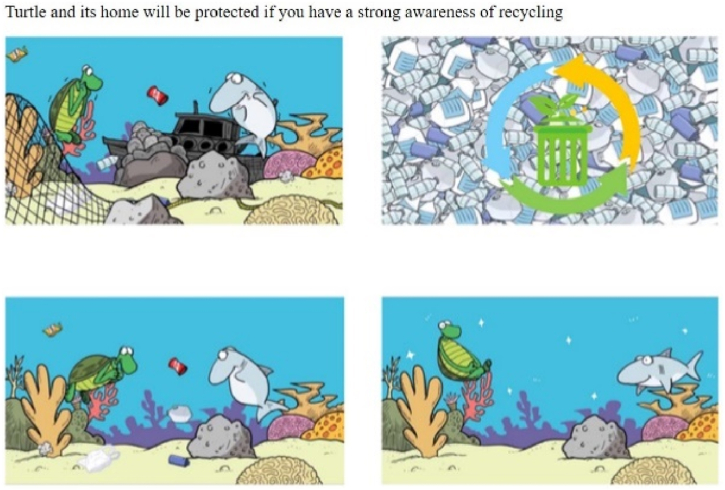
Positively framed cartoon with a turtle.

**Fig. 2:**
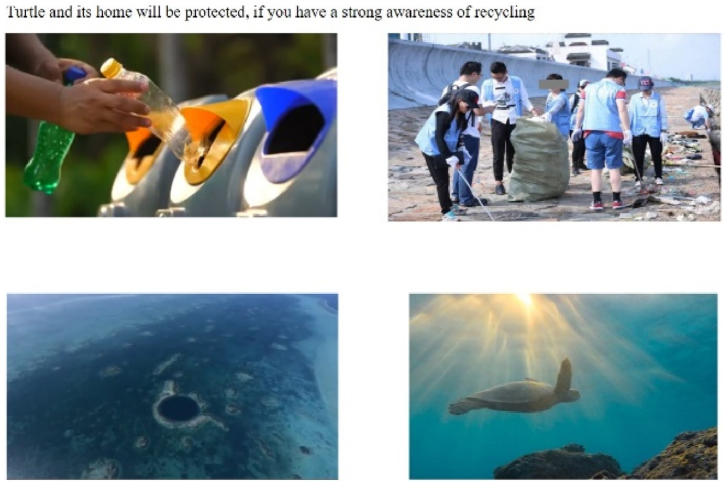
Positively framed photograph with a turtle.

**Fig. 3:**
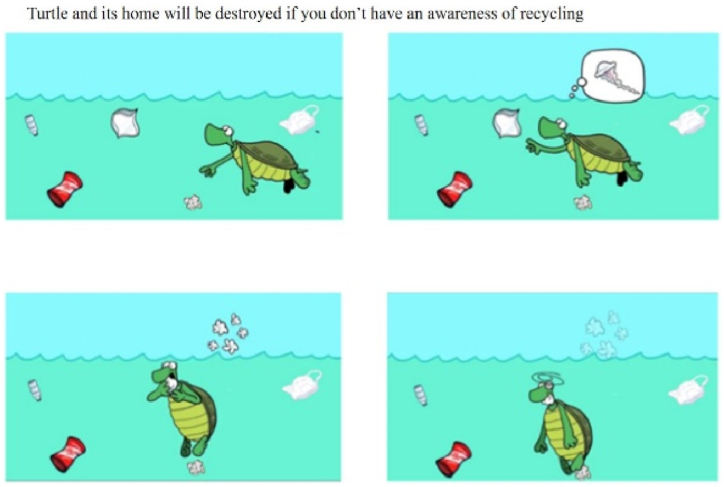
Negatively framed cartoon with a turtle.

**Fig. 4:**
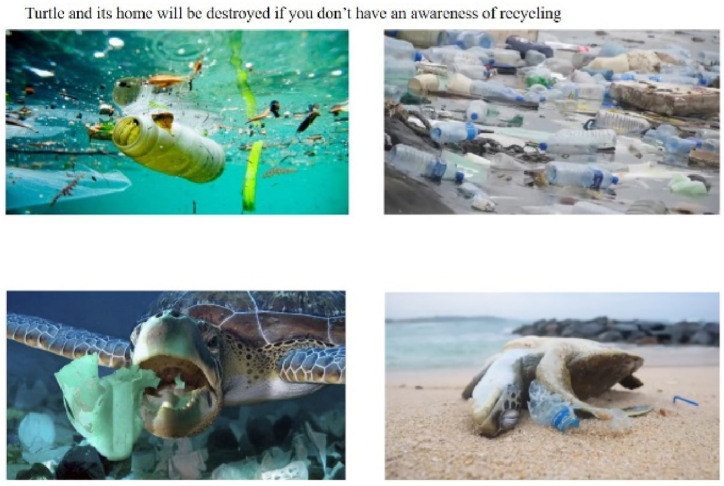
Negatively framed photograph with a turtle.

### 3.2 Samples and data collections

Data collection and message exposure were conducted via an online platform in Mainland China (wjx.cn) from April 2022 to July 2022. Statistical analyses were performed using IBM SPSS 26.0 (IBM Corp), with 95 % confidence intervals applied across the eight experimental conditions. Participants were recruited from several public colleges and high schools in coastal regions of China, volunteering and receiving a reward after the experiments. This study comprised two parts. The first part checked the effectiveness of the designed pictures. The participants were asked to indicate whether the message was positively or negatively framed. In addition, relevant personal information, such as age, gender, and educational background, was collected (see Table 1). In the second part, participants responded to questions regarding the independent variables, namely preference, and SE. For the specific questions, see Table 2.

**Table 1:** Demographic profile.

Characteristics	N	Percentage
Gender		
Male	105	46.26 %
Female	122	53.74 %
Age		
18–22 years	179	78.85 %
23–30 years	45	19.82 %
31–35	3	1.3 %
Education		
Undergraduate	179	78.85 %
Graduate	46	20.26 %
Doctor	2	0.88 %

**Table 2:** Measurement of variables and questions.

Variable	Measurement Questions
1: Preference	(Q i) How appealing is it?
	(Q ii) How strong an emotional response did the design evoke?
Cronbach's a>0.75	(Q iii) How much did you like this design?
2: Garbage sorting-related self-efficacy	(Q iv) I believe I don't throw garbage when I travel to a beach.
	(Q v) I know how to collect garbage when I visit the seaside.
Cronbach's a>0.75	(Q vi) I hope I can do something for the future by not littering.

Before data analysis, G*Power software was used to compute the required sample size, resulting in a target of approximately 128 participants. Thus, 240 respondents were deemed sufficient for the study. The selected participants were randomly assigned to one of eight message conditions. Thirteen participants were excluded due to missing data, outliers, or inconsistencies, leaving 227 valid participants. Among them, 112 were assigned to the positive condition and 115 to the negative condition. Most respondents were young adults, with 53.74 % being female. Table 1 shows the demographic profile of the participants.

### 3.3 Measurement of outcome variables

This study measured the communication effect of the messages using two outcome variables: preference for the advertisement and the viewer's self-efficacy. Viewers' attitudes toward the advertisement were assessed using three questions adapted from Bao et al. [44], each rated on a 5-point scale ranging from 1 (I disagree) to 5 (I agree). Self-efficacy was measured using three questions adapted from Updegraffa et al. and made some revisions [45], also rated on a 5-point Likert scale. For detailed information, see Table 2.

## 4 Results

### 4.1 Manipulation check

The question regarding whether the message was clearly expressed was designed to test whether the framing effect, narrative image types, and psychological distance had been successfully designed. An independent-sample *t*-test was used to determine if the framing effect was successfully manipulated. The results indicated a significant difference between positive and negative framing (M positive = 4.224, M negative = 3.001, t = 9.118, p < 0.05).

### 4.2 Hypothesis testing

A three-way analysis of variance (ANOVA) was conducted with narrative image types, message framing, and psychological distance as independent variables, and preference and self-efficacy as dependent variables. The results showed significant main effects of image types [F (1, 227) = 44.351, p < 0.05] and message framing [F (1, 227) = 8.728, p < 0.05] on preference. Additionally, there was a significant interaction effect between image types and message framing on preference [F (1, 227) = 5.977, p < 0.05]. Regarding self-efficacy, the main effect of image types was significant [F (1, 227) = 5.489, p < 0.05], as was the interaction effect between image types and message framing [F (1, 227) = 7.524, p < 0.01]. The interactions between image types and psychological distance [F (1, 227) = 9.013, p < 0.05] and between message framing and psychological distance [F (1, 227) = 6.784, p < 0.05] were also observed to be significant. Last but not least, there was a marginally statistically significant three-way interaction among message framing, psychological distance, and narrative image types [F (1, 227) = 3.293, p < 0.1]. The detailed results are shown in Table 3.

**Table 3:** Effects on advertisement preference and self-efficacy.

Sources of Variation	Preference		Self-Efficacy	
	F Value η2		F Value η2	
Narrative image types (NIT)	44.351^c^	0.168	5.489^b^	0.024
Message framing (MF)	8.728^b^	0.038	0.005	0.000
Psychological distance (PD)	3.282	0.015	2.042	0.009
NIT × MF	5.977^b^	0.027	7.524^b^	0.033
NIT × PD	0.093	0.000	9.013^b^	0.040
MF × PD	0.178	0.001	6.784^b^	0.030
NIT × MF × PD	0.676	0.003	3.293^a^	0.015

Note.

a *p* < 0.1.

b *p* < 0.05.

c *p* < 0.001.

**Fig. 5:**
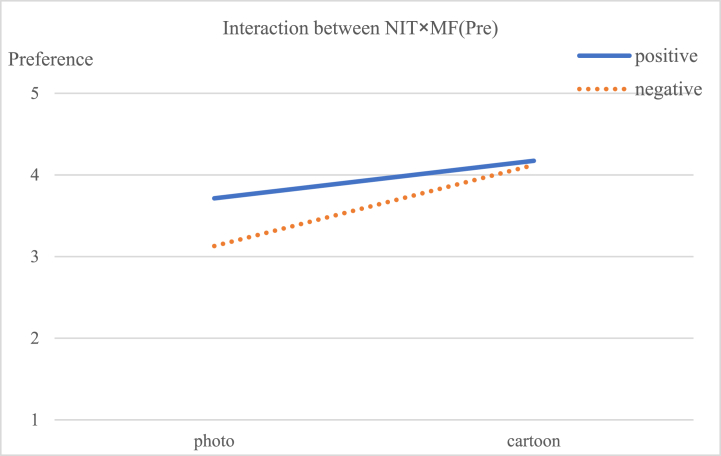
Interaction of narrative image types × message framing on preference.

Viewers are likely to have a stronger preference (M cartoon = 4.146, M photo = 3.421, [F (1, 227) = 44.351, p < 0.001] for messages and greater self-efficacy (M cartoon = 4.106, M photo = 3.907, [F(1, 227) = 28.435, p < 0.05] when the messages are presented as cartoons, compared with photographs. Message framing had a main effect on preference [F (1, 227) = 8.728, p < 0.05], but there was no significant effect on viewers’ self-efficacy [F (1, 227) = 0.005, p = 0.944]. The interaction effect of image types × message framing on message preference [F (1, 227) = 5.977, p < 0.05] and self-efficacy [F (1, 227) = 7.524, p < 0.05] both reached the significance level. Specifically, when presented in a positive frame, cartoons generated stronger preference (M photos = 3.713, M cartoon = 4.172, p < 0.05) and stronger self-efficacy than photos (M photos = 3.791, M cartoon = 4.213, p < 0.05). Therefore, Hypothesis 1 was supported. The simple interactive results of narrative image types and message framing are shown in Table 4, and Fig. 5.

**Table 4:** Dependent measures across message framing and narrative image types.

Independent Variables	Message Framing	Image Types	Mean	SD	I-J	p
Preference (Pre)	Positive	Photo (I)	3.713	0.785	−0.458	0.003
		Cartoon (J)	4.172	0.798	0.458	
	Negative	Photo (I)	3.129	0.876	−0.990	0.000
		Cartoon (J)	4.121	0.813	0.990	
Self-efficacy (SE)	Positive	Photo (I)	3.791	0.764	−0.418	0.000
		Cartoon (J)	4.213	0.624	0.418	
	Negative	Photo (I)	4.022	0.594	0.033	0.776
		Cartoon (J)	3.999	0.573	−0.033

**Fig. 6:**
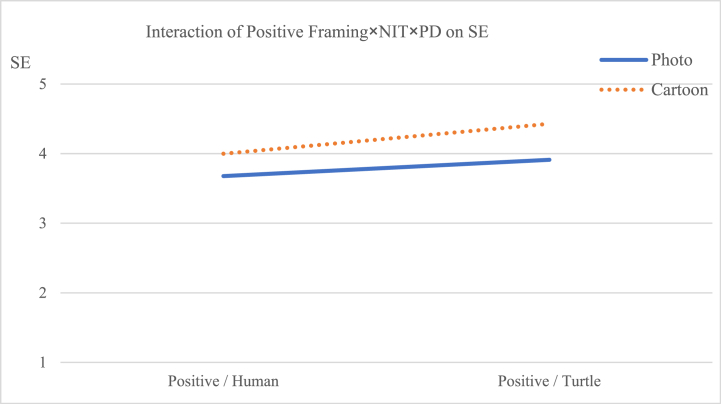
Interactive effects of positive framing × narrative image types × psychological distance on viewers' self-efficacy.

**Fig. 7:**
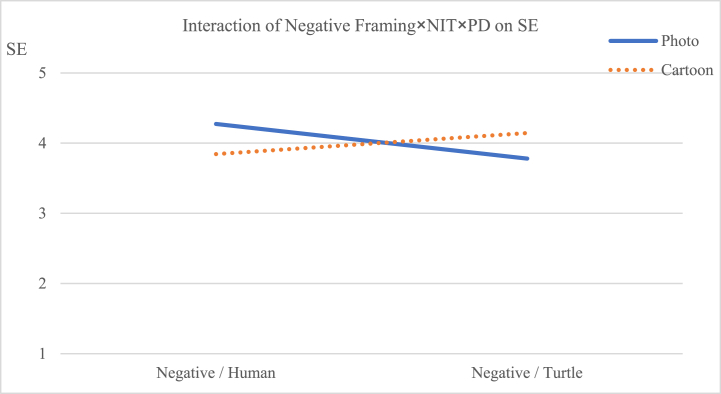
Interactive effects of negative framing × narrative image types × psychological distance on viewers' self-efficacy.

The interaction effect of message framing × psychological distance on preference [F (1, 227) = 0.178, *p* = 0.673] did not reach significance, but the effect on self-efficacy [F (1, 227) = 6.784, *p* < 0.05] reached significance. The three-way interaction among image types, message framing, and psychological distance did not have a statistically significant effect on preference [F (1, 227) = 0.676, p = 0.412], but it had a statistically significant effect on self-efficacy [F (1, 227) = 3.293, p < 0.1]. The follow-up ANOVA revealed that when the frame was positive, viewers were likely to engender stronger self-efficacy through information with turtles accompanied by cartoons than those with photographs (M photo × turtle = 3.912, M cartoon × turtle = 4.429, [F (1, 227) = 9.541, *p* < 0.01]; see Table 5 and Fig. 6). Therefore, Hypothesis 2 was supported. On the contrary, when the frame was negative, viewers were likely to engender stronger self-efficacy through information with humans accompanied by photos than those with cartoons (M photo × human = 4.273, M cartoon × human = 3.844, [F (1, 227) = 6.720, *p* < 0.05]; see Table 5 and Fig. 7).

**Table 5:** Dependent measures for the effects of narrative image types, message framing and psychological distance on SE.

SE	Positive				Negative			
	Human		Turtle		Human		Turtle	
	Photo	Cartoon	Photo	Cartoon	Photo	Cartoon	Photo	Cartoon
Mean	3.678	3.999	3.912	4.429	4.273	3.844	3.780	4.144
SD	0.919	0.545	0.544	0.634	0.537	0.563	0.552	0.553
F	3.818		9.541		6.720		5.073	
P	0.052		0.002		0.010		0.025

**Fig. 8:**
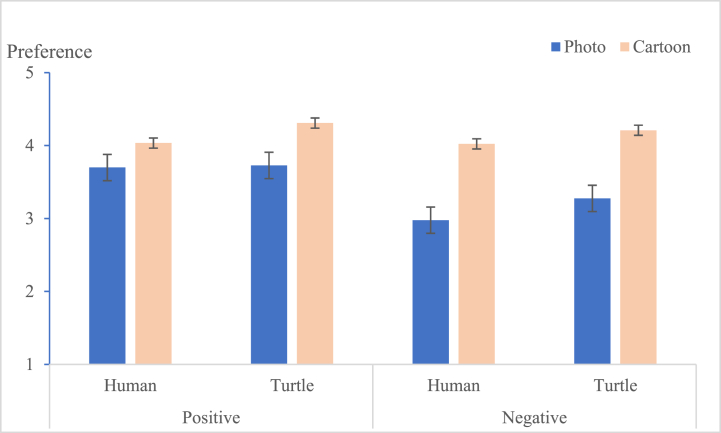
The preference for the design of eight groups.

**Fig. 9:**
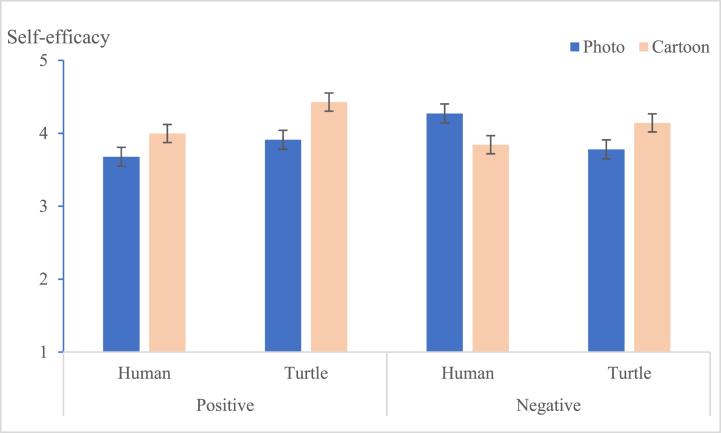
The perceived self-efficacy engendered by the eight groups.

The distribution of preferred designs is summarized in Fig. 8. As a result, no matter whether the framing was positive or negative, the cartoon with the turtle design was the most favorite. Meanwhile, the distribution for viewers' self-efficacy is summarized in Fig. 9. When the message was positive and the characters were marine animals (turtles), it engendered the most self-efficacy in the viewers in the eight groups, but when the message was negative and the characters were human, viewers’ self-efficacy was higher than for those with cartoons.

### 4.3 Regression analysis

To evaluate the relationship between viewers' preference towards a message and self-efficacy, that is, whether or not preference can be used to predict self-efficacy, we used regression analysis in SPSS 26.0. The independent variable was preference; self-efficacy was considered as the dependent variable. From Table 6, F = 93.152, P = 0.000 < 0.05, it was statistically significant, indicating that the relationship of the regression equation was significant. This suggested that preference was a significant factor influencing viewers’ self-efficacy. Hypothesis 3 was thus supported.

**Table 6:** Regression analysis between preference and self-efficacy.

Variation	*β*	*R*^*2*^	*t*	*p*	*F*
Preference	0.541	0.293	9.652	0.000	93.152

## 5 Discussion

This study discusses different design strategies to encourage young tourists to recycle, not litter. More specifically, this study explored the interaction of narrative image types, message framing, and psychological distance. The results showed that: (1) Narrative image types had a main effect on viewers' preferences and SE. (2) Psychological distance had no main effect on preference and SE. (3) The interaction effect of narrative image types and message framing on viewers’ preferences and SE were equally significant. (4) Narrative image types, message framing, and psychological distance had a three-way interactive effect on SE.

The common phrase “a picture is worth a thousand words” underscores the importance of visual communication, especially in modern times [23]. Our results indicate that young people are particularly engaged by cartoons, confirming previous studies that ecologically oriented cartoons effectively persuade the public to protect the natural world [46]. Compared to photos, cartoons are more successful in overcoming resistance to persuasion, especially among young people, and significantly enhance awareness of marine protection. Therefore, cartoons are validated as an effective educational tool in marine conservation communication (MCC).

Positively framed messages were found to encourage a more positive attitude towards marine environmental protection and generate greater interest than negatively framed messages. This aligns with previous studies that positive descriptive norms and imagery elicit more positive emotions [18]. However, our study found no significant effect of message framing on SE. Regardless of whether the framing was positive (e.g., turtles swimming freely in the ocean, people living in harmony with nature) or negative (e.g., dead turtles, a girl surrounded by garbage), the effect on SE remained unchanged. This suggests that SE depends on multiple factors, such as risk perception [47], subjective knowledge, and the perception of harmful consequences [48], rather than framing alone.

Psychological distance, while important for pro-environmental behaviors, showed no clear effect on preference or SE in our study. Related studies have shown that the proximally framed information in information construction increased participants’ engagement with environmental protection, affectively, cognitively, and behaviorally. However, our results, at first glance, seem disappointing. There was no clear effect on either preference or SE, i.e., there was no difference between the proximal (human) and the distant framing (turtle). Further research revealed that this result is not unexpected. The advantage of proximal psychology is not evident when psychological distance is considered alone. In other words, proximity alone does not increase engagement [35].

The interaction between message framing and image types was significant [19], suggesting that congruent combinations are more effective. Cartoons, in particular, scored higher than photographs in terms of preference. The visually engaging cartoons depicted a harmonious ocean environment and its consequences. In the negatively framed message, the gloomy color, exaggerated expressions, and skeletons in the mountains of plastic waste create a sad tone. In contrast, positively framed messages use colorful tones and happy expressions to create a joyful atmosphere. As young people tend to pursue pleasure, positively framed messages are more attractive. Photographs, mostly screenshots from expository documentaries, were neutral and objective [49], thus eliciting a weaker emotional response compared to cartoons. Overall, these findings highlight the importance of using engaging and emotionally resonant visual narratives to enhance the effectiveness of environmental communication among young audiences.

We found that the interaction among image types, message framing, and psychological distance significantly affected on SE, particularly when participants viewed a positively framed message featuring a turtle cartoon form. Previous studies suggest that connecting with viewers requires delivering the right message in the right format to the right audience. Several factors likely explain this result. First, the participants were young, a demographic known for its eagerness to learn and open-mindedness. Young people are imaginative and independent thinkers, and cartoons, with their colorful and creative elements, cater to their curiosity. Our findings suggest that young people prefer cartoons, possibly because they resonate more with their imaginative nature. Additionally, using sea turtles and whales in these messages fosters a closer relationship between viewers and these animals. Viewers become aware that their attitudes and behaviors directly impact the survival of these creatures, engaging their compassion and sense of responsibility [50]. Consequently, viewers are emotionally engaged by persuasive messages delivered through these animals and are motivated to change their behavior.

### 5.1 Theoretical implications

This study addresses the issue of garbage recycling by employing common information construction methods to raise awareness about marine environmental protection among young tourists and influence their behavior. Our experimental results indicate that cartoons featuring sea turtles, especially when presented with positive outcomes, are the most effective means of conveying marine conservation messages to young people. In other words, positive information depicted through marine animals can enhance young people's preference for the information and boost their self-efficacy (SE) in marine protection. This approach allows them to enjoy their travels without compromising their sense of relaxation. Therefore, cartoons are an effective tool for disseminating marine conservation information, given the synergistic effects of message framing and psychological distance.

### 5.2 Practical implications

Based on our experimental findings, we recommend that communications designed to persuade young tourists not to litter should employ a combination of animal imagery, positive message framing, and cartoon format for screen presentations. Given the preferences of young people in terms of MMC, this approach is likely to be highly effective.

### 5.3 Limitations and future research

Our research has several limitations. Firstly, the participants were young people, who typically prefer nature-themed cartoons. This preference may not extend to other demographic groups, such as older adults. Future studies should replicate this research with different age groups, demographics, and geographic contexts to broaden the applicability of the findings. Secondly, the aesthetic quality of the images used in this study, including color matching and animal image drawing, could be improved. Future research should specifically examine these aesthetic aspects to enhance the effectiveness of information construction. Notably, different images can elicit varying emotional responses [20^,^52]. For instance, in the negative framing scenario, a dead turtle is shown in photographs. However, depicting dead humans might induce fear [51], so a photograph of people surrounded by garbage was presented. The emotional responses to these images may differ: a dead turtle might evoke sadness [52], while a girl surrounded by garbage might cause nausea and disgust, thus influencing viewer's judgments and decisions. Future research should account for these emotional responses to ensure consistency. Lastly, while preference and SE are critical components of behavioral change according to social cognitive theory [13], they are indirect measures of persuasion [51]. Future studies should include follow-up monitoring and other research methods to enhance the reliability of the findings.

## 6 Conclusion

This study examined the effects of narrative image types, message framing, and psychological distance on marine protection to guide the design of persuasive communication. Our findings indicate that the combination of image types, message framing, and psychological distance had a three-way interactive effect on self-efficacy. Specifically, cartoons featuring turtles and positive outcomes are the most effective way to communicate messages about marine garbage recycling to young people. Additionally, young people's preference for such information enhances their self-efficacy in marine garbage recycling efforts. These results underscore the importance of using cartoons in information campaigns in garbage recycling, particularly in materials targeted at youth, such as university campus posters or online pop-ups for college students. To effectively boost the self-efficacy of young individuals in marine environmental protection, it is essential to use friendly and positive imagery while avoiding depictions of brutality and suffering.

## Data availability statement

Data are contained within the article.

## Funding

This research was supported by the 10.13039/501100001809National Natural Science Foundation of China (No:52005251), the 10.13039/501100018562Social Science Foundation of Jiangsu Province (No.20YSC011), Education Reform Project from Nanjing Tech University (20210257), and 2018 Jiangsu Province graduate scientific research innovation project (KYCX18_0069), and the general Project of Philosophy and Social Science Research in Colleges and Universities in Jiangsu Province (2023SJYB0211).

## Informed consent statement

The study was reviewed and approved by Nanjing Tech University, with the approval number: NJTECH-1-4. All participants provide written informed consent to participate in the study.

## CRediT authorship contribution statement

**Mo Chen:** Writing – review & editing, Writing – original draft, Validation, Supervision, Software, Resources, Methodology, Investigation, Funding acquisition, Formal analysis, Conceptualization. **Kexin Chen:** Writing – review & editing, Software, Methodology, Investigation. **Yang Qin:** Writing – review & editing, Writing – original draft, Software, Methodology, Investigation. **Yanfei Zhu:** Supervision, Methodology, Investigation, Funding acquisition, Formal analysis, Conceptualization.

## Declaration of competing interest

The authors declare that they have no known competing financial interests or personal relationships that could have appeared to influence the work reported in this paper.

Mo Chen reports financial support was provided by 10.13039/501100001809National Natural Science Foundation of China, China (No.52005251). Mo Chen reports financial support was provided by 10.13039/501100018562Social Science Foundation of Jiangsu Province, China (No.20YSC011). Mo Chen reports financial support was provided by General Project of Philosophy and Social Science Research in Colleges and Universities in Jiangsu Province, China (No.2023SJYB0211). If there are other authors, they declare that they have no known competing financial interests or personal relationships that could have appeared to influence the work reported in this paper.

## Figures and Tables

**Figure:**
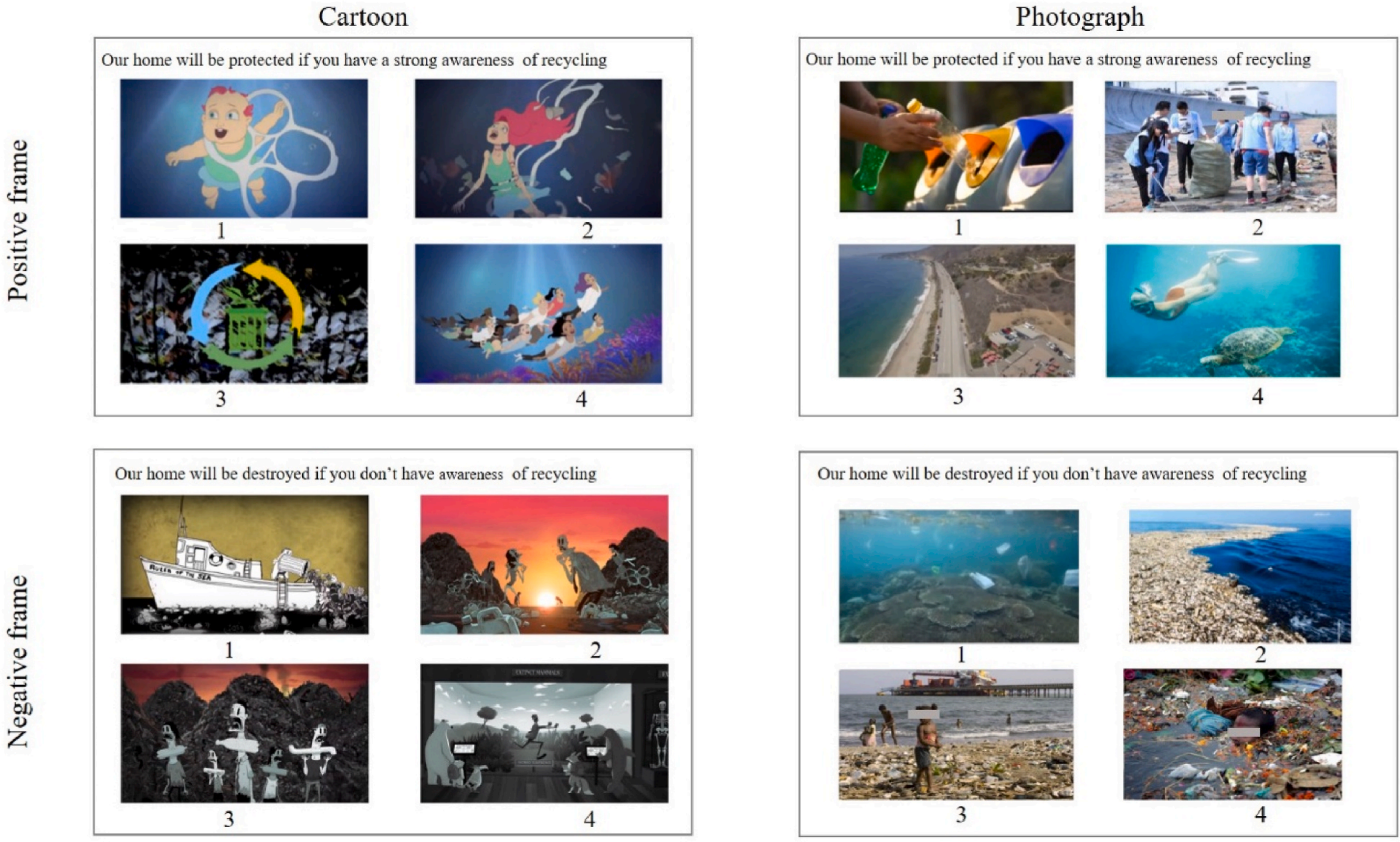


**figs1:**
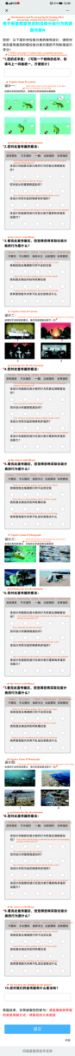


**figs2:**
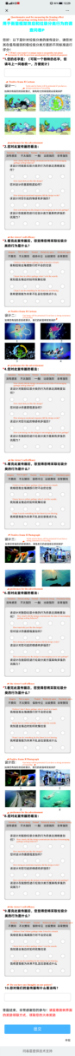


## References

[1] Ramos B de, Alencar M.V., Rodrigues F.L. (2021). Spatio-temporal characterization of litter at a touristic sandy beach in South Brazil. Environ. Pollut..

[2] García-Gómez J.C., Garrigós M., Garrigós J. (2021). Plastic as a vector of dispersion for marine species with invasive potential. A review. Frontiers in Ecology and Evolution.

[3] Hayati Y., Adrianto L., Krisanti M. (2020). Magnitudes and tourist perception of marine debris on small tourism island: assessment of Tidung Island, Jakarta, Indonesia. Mar. Pollut. Bull..

[4] Kerber H., Kramm J. (2021). On‐ and offstage: encountering entangled waste–tourism relations on the Vietnamese Island of Phu Quoc. Geogr. J..

[5] Manomaivibool P. (2015). Wasteful tourism in developing economy? A present situation and sustainable scenarios. Resour. Conserv. Recycl..

[6] Hartley J.M., Stevenson K.T., Peterson M.N. (2021). Intergenerational learning: a recommendation for engaging youth to address marine debris challenges. Mar. Pollut. Bull..

[7] Barboza L.G.A., Dick Vethaak A., Lavorante B.R.B.O. (2018). Marine microplastic debris: an emerging issue for food security, food safety and human health. Mar. Pollut. Bull..

[8] Campbell Marnie L., Chloe Paterson D., Kinslow Amber (2014). Littering dynamics in a costal industrial setting: the influence of non-resident populations.

[9] Šaparnienė D., Mejerė O., Raišutienė J. (2022). Expression of behavior and attitudes toward sustainable tourism in the youth population: a search for statistical types. Sustainability.

[10] Veiga J.M., Vlachogianni T., Pahl S. (2016). Enhancing public awareness and promoting co-responsibility for marine litter in Europe: the challenge of MARLISCO. Mar. Pollut. Bull..

[11] Bandura A. (1982). Self-efficacy mechanism in human agency. Am. Psychol..

[12] Bandura A. (1984). Recycling misconceptions of perceived self-efficacy. Cognit. Ther. Res..

[13] Bandura A. (1977). Self efficacy toward a unifying theory of behavioral change.

[14] Zhu Y., Wang Y., Li Y. (2022). The construction of image reference points and text appeals information tailoring in promoting diners' public environment maintenance behavior intention. Int. J. Environ. Res. Publ. Health.

[15] Goldstein N.J., Cialdini R.B., Griskevicius V. (2008). A room with a viewpoint: using social norms to motivate environmental conservation in hotels. J. Consum. Res..

[16] Claudet J., Bopp L., Cheung W.W.L. (2020). A roadmap for using the UN decade of ocean science for sustainable development in support of science, policy, and action. One Earth.

[17] Botsis T., Fairman J.E., Moran M.B. (2020). Visual storytelling enhances knowledge dissemination in biomedical science. J. Biomed. Inf..

[18] Poškus M.S., Pilkauskaitė Valickienė R., Kuzinas A. (2019). The effects of descriptive imagery and appeals on emotions and intentions related to pro-environmental behavior. Sustainability.

[19] Septianto F., Lee M.S.W. (2020). Emotional responses to plastic waste: matching image and message framing in encouraging consumers to reduce plastic consumption. Australas. Market J..

[20] Erskine E., Baillie R., Lusseau D. (2021). Marine Protected Areas provide more cultural ecosystem services than other adjacent coastal areas. One Earth.

[21] Shin Y., Noone B.M., Robson S.K.A. (2020). An exploration of the effects of photograph content, photograph source, and price on consumers' online travel booking intentions. J. Trav. Res..

[22] Pan B., Zhang L., Law R. (2013). The complex matter of online hotel choice. Cornell Hospitality Quarterly.

[23] Small E. (2016). The value of cartoons for biodiversity conservation. Biodiversity.

[24] Katoppo M.L., Irwandi E., Ng A.H. (2020). Building environmental awareness for future generation through educational comic: the story of 4 th -grade students Darussalam elementary school, Panongan, Tangerang. IOP Conf. Ser. Earth Environ. Sci..

[25] Sukri A., Efendi I., Hastuti R. (2020). The effect of coral reef comic media implementation on students' environmental care attitude in Indonesia. J. Phys. Conf..

[26] Kolandai‐Matchett K., Armoudian M., Thrush S. (2021). Communicating complex marine science: does media format matter?. Aquat. Conserv. Mar. Freshw. Ecosyst..

[27] Komatsu H., Kubota H., Tanaka N. (2020). Designing information provision to serve as a reminder of altruistic benefits: a case study of the risks of air pollution caused by industrialization. PLoS One.

[28] O'Neill S., Nicholson-Cole S. (2009). Fear won't do it. Sci. Commun..

[29] Wilson S.P., Verlis K.M. (2017). The ugly face of tourism: marine debris pollution linked to visitation in the southern Great Barrier Reef, Australia. Mar. Pollut. Bull..

[30] Jacobson S.K., Morales N.A., Chen B. (2019). Love or Loss: effective message framing to promote environmental conservation. Appl. Environ. Educ. Commun. Int. J..

[31] O'Keefe D.J., Jensen J.D. (2009). The relative persuasiveness of gain-framed and loss-framed messages for encouraging disease detection behaviors: a meta-analytic review. J. Commun..

[32] Stillman P.E., Fujita K., Sheldon O. (2018). From “me” to “we”: the role of construal level in promoting maximized joint outcomes. Organ. Behav. Hum. Decis. Process..

[33] Trope Y., Liberman N. (2010). Construal-level theory of psychological distance. Psychol. Rev..

[34] Shih T.-J., Lin C.-Y. (2017). Developing communication strategies for mitigating actions against global warming: linking framing and a dual processing model. Environmental Communication.

[35] Brügger A., Morton T.A., Dessai S. (2016). “Proximising” climate change reconsidered: a construal level theory perspective. J. Environ. Psychol..

[36] Lu H., Siemer W.F., Baumer M.S. (2018). Exploring the role of gain versus loss framing and point of reference in messages to reduce human–bear conflicts. Soc. Sci. J..

[37] Froehlich J., Findlater L., Landay J. (2010). Proceedings of the 28th International Conference on Human Factors in Computing Systems.

[38] Wang Y., Dai Y., Li H. (2021). Social media and attitude change: information booming promote or resist persuasion?. Front. Psychol..

[39] Hirsh J.B., Kang S.K., Bodenhausen G.V. (2012). Personalized persuasion: tailoring persuasive appeals to recipients' personality traits. Psychol. Sci..

[40] Jugert P., Greenaway K.H., Barth M. (2016). Collective efficacy increases pro-environmental intentions through increasing self-efficacy. J. Environ. Psychol..

[41] Lauren N., Fielding K.S., Smith L. (2016). You did, so you can and you will: self-efficacy as a mediator of spillover from easy to more difficult pro-environmental behaviour. J. Environ. Psychol..

[42] Shafiei A., Maleksaeidi H. (2020). Pro-environmental behavior of university students: application of protection motivation theory. Global Ecology and Conservation.

[43] Huang C. (2016). Achievement goals and self-efficacy: a meta-analysis. Educ. Res. Rev..

[44] Bao Q., Shaukat M.M., Elantary A., Et A. (2016).

[45] Updegraff J.A., Sherman D.K., Luyster F.S. (2007). The effects of message quality and congruency on perceptions of tailored health communications. J. Exp. Soc. Psychol..

[46] Brown W.J., Lindvall T.R. (2019). Green cartoons: toward a pedagogy of the animated parable. Animation.

[47] Bruijn G-J de, Spaans P., Jansen B. (2016). Testing the effects of a message framing intervention on intentions towards hearing loss prevention in adolescents. Health Educ. Res..

[48] Ai P., Li W., Yang W. (2021). Adolescents' social media use and their voluntary garbage sorting intention: a sequential mediation. Model.

[49] Bradbury J.D., Guadagno R.E. (2020). Documentary narrative visualization: features and modes of documentary film in narrative visualization. Inf. Visual..

[50] Swim J.K., Bloodhart B. (2015). Portraying the perils to polar bears: the role of empathic and objective perspective-taking toward animals in climate change communication. Environmental Communication.

[51] Ma Z. (2021).

[52] Iyer A., Webster J., Hornsey M.J. (2014). Understanding the power of the picture: the effect of image content on emotional and political responses to terrorism. J. Appl. Soc. Psychol..

