# Supplementary material for: A study on the effectiveness of narrative image types, message framing, and psychological distance in enhancing young people's self-efficacy in marine garbage recycling
Source: Heliyon. 2024 Jul 21;10(15):e34919. doi: 10.1016/j.heliyon.2024.e34919 (PMC11336297; doi:10.1016/j.heliyon.2024.e34919)
Supplement: Multimedia component 1 [file mmc1.pdf]

Preference ANOVA

```
UNIANOVA score BY framepln2 psychologyhlt2 typeplc2
  /METHOD=SSTYPE(3)
  /INTERCEPT=INCLUDE
  /POSTHOC=framepln2 psychologyhlt2 typeplc2(LSD)
  /PLOT=PROFILE(framepln2*psychologyhlt2*typeplc2)
  /EMMEANS=TABLES(framepln2) COMPARE ADJ(LSD)
  /EMMEANS=TABLES(psychologyhlt2) COMPARE ADJ(LSD)
  /EMMEANS=TABLES(typeplc2) COMPARE ADJ(LSD)
  /EMMEANS=TABLES(framepln2*psychologyhlt2)
  /EMMEANS=TABLES(framepln2*typeplc2)
  /EMMEANS=TABLES(psychologyhlt2*typeplc2)
  /EMMEANS=TABLES(framepln2*psychologyhlt2*typeplc2)
  /PRINT=ETASQ DESCRIPTIVE
  /CRITERIA=ALPHA(.05)
  /DESIGN=framepln2 psychologyhlt2 typeplc2 framepln2*psychologyhlt2 framepln2*typeplc2
    psychologyhlt2*typeplc2 framepln2*psychologyhlt2*typeplc2.
```

方差的单变量分析 ➔ One-Way ANOVA

备注

已创建输出		28-APR-2024 15:15:14
注释		
输入	活动数据集	数据集0
	过滤器	<无>
	权重	<无>
	拆分文件	<无>
	工作数据文件中的行数	227
缺失值处理	对缺失的定义	将用户定义的缺失值视为缺失。
	使用的个案数	统计基于所有那些对于模型中的所有变量都具有有效数据的个案。

语法		UNIANOVA score BY framep1n2 psychologyh1t2 typep1c2<br>/METHOD=SSTYPE(3)<br>/INTERCEPT=INCLUDE<br>/POSTHOC=framep1n2 psychologyh1t2 typep1c2(LSD)<br><br>/PLOT=PROFILE(framep1n2*psychologyh1t2*typep1c2)<br>/EMMEANS=TABLES(framep1n2) COMPARE ADJ(LSD)<br>/EMMEANS=TABLES(psychologyh1t2) COMPARE ADJ(LSD)<br>/EMMEANS=TABLES(typep1c2) COMPARE ADJ(LSD)<br><br>/EMMEANS=TABLES(framep1n2*psychologyh1t2)<br><br>/EMMEANS=TABLES(framep1n2*typep1c2)<br><br>/EMMEANS=TABLES(psychologyh1t2*typep1c2)<br><br>/EMMEANS=TABLES(framep1n2*psychologyh1t2*typep1c2)<br>/PRINT=ETASQ DESCRIPTIVE<br>/CRITERIA=ALPHA(.05)<br>/DESIGN=framep1n2 psychologyh1t2 typep1c2 framep1n2*psychologyh1t2 framep1n2*typep1c2<br>psychologyh1t2*typep1c2 framep1n2*psychologyh1t2*typep1c2.
资源	处理程序时间	00:00:04.62
	耗用时间	00:00:01.23

[数据集0]

主体间因子 ➔ Between-Subjects Factors

		个案数
framepln2	1.00	112
	2.00	115
psychologyhlt2	1.00	113
	2.00	114
typeplc2	1.00	113
	2.00	114

描述统计 ➡ Descriptive Statistics

因变量: score

framepln2	psychologyhlt2	typeplc2	平均值	标准偏差	个案数
1.00	1.00	1.00	3.6999	.68699	29
		2.00	4.0357	.96583	28
		总计	3.8649	.84524	57
	2.00	1.00	3.7284	.89173	27
		2.00	4.3095	.57326	28
		总计	4.0242	.79570	55
	总计	1.00	3.7136	.78510	56
		2.00	4.1726	.79897	56
		总计	3.9431	.82150	112
2.00	1.00	1.00	2.9773	.74173	28
		2.00	4.0238	1.00176	28
		总计	3.5005	1.02055	56
	2.00	1.00	3.2764	.97987	29
		2.00	4.2106	.59122	30
		总计	3.7514	.92743	59
	总计	1.00	3.1295	.87641	57
		2.00	4.1204	.81367	58
		总计	3.6292	.97773	115
总计	1.00	1.00	3.3449	.79629	57
		2.00	4.0298	.97499	56
		总计	3.6843	.94981	113
	2.00	1.00	3.4943	.95741	56
		2.00	4.2583	.57965	58
		总计	3.8830	.87338	114
	总计	1.00	3.4190	.87909	113
		2.00	4.1461	.80333	114
		总计	3.7841	.91564	227

主体间效应检验 ➡ Tests of Between-Subjects Effects

因变量: score

源	III 类平方和	自由度	均方	F	显著性	偏 Eta 平方
修正模型	42.559[a]	7	6.080	9.063	.000	.225
截距	3245.166	1	3245.166	4837.280	.000	.957
framepln2	5.856	1	5.856	8.728	.003	.038
psychologyhlt2	2.202	1	2.202	3.282	.071	.015
typeplc2	29.753	1	29.753	44.351	.000	.168
framepln2 * psychologyhlt2	.119	1	.119	.178	.673	.001
framepln2 * typeplc2	4.010	1	4.010	5.977	.015	.027
psychologyhlt2 * typeplc2	.063	1	.063	.093	.760	.000
framepln2 * psychologyhlt2 * typeplc2	.453	1	.453	.676	.412	.003
误差	146.920	219	.671			
总计	3440.005	227				
修正后总计	189.478	226				

a. R 方 = .225（调整后 R 方 = .200）

估算边际平均值 ➡ Estimated Marginal Means

1. framepln2

估算值 ➡ Estimates

因变量: score

framepln2	平均值	标准误差	95% 置信区间	
			下限	上限
1.00	3.943	.077	3.791	4.096
2.00	3.622	.076	3.471	3.773

成对比较 ➡ Pairwise Comparisons

因变量:　score

(I) framepln2	(J) framepln2	平均值差值 (I-J)	标准误差	显著性[b]	差值的 95% 置信区间[b]	
					下限	上限
1.00	2.00	.321*	.109	.003	.107	.536
2.00	1.00	-.321*	.109	.003	-.536	-.107

基于估算边际平均值

*. 平均值差值的显著性水平为 .05。

b. 多重比较调节：最低显著差异法（相当于不进行调整）。

单变量检验 ➡ Univariate Tests

因变量:　score

	平方和	自由度	均方	F	显著性	偏 Eta 平方
对比	5.856	1	5.856	8.728	.003	.038
误差	146.920	219	.671			

F 检验 framepln2 的效应。此检验基于估算边际平均值之间的线性无关成对比较。

2. psychologyh1t2

估算值 ➡ Estimates

因变量:　score

psychologyh1t2	平均值	标准误差	95% 置信区间	
			下限	上限
1.00	3.684	.077	3.532	3.836
2.00	3.881	.077	3.730	4.033

成对比较 ➡ Pairwise Comparisons

因变量:　score

(I) psychologyh1t2	(J) psychologyh1t2	平均值差值 (I-J)	标准误差	显著性[a]	差值的 95% 置信区间[a]	
					下限	

1.00	2.00	-.197	.109	.071	-.411	
2.00	1.00	.197	.109	.071	-.017	

成对比较 ➔ Pairwise Comparisons

因变量: score

(I) psychologyhlt2	(J) psychologyhlt2	差值的 95% 置信区间
		上限
1.00	2.00	.017
2.00	1.00	.411

基于估算边际平均值

a. 多重比较调节：最低显著差异法（相当于不进行调整）。

单变量检验 ➔ Univariate Tests

因变量: score

	平方和	自由度	均方	F	显著性	偏 Eta 平方
对比	2.202	1	2.202	3.282	.071	.015
误差	146.920	219	.671			

F 检验 psychologyhlt2 的效应。此检验基于估算边际平均值之间的线性无关成对比较。

3. typeplc2

估算值 ➔ Estimates

因变量: score

typeplc2	平均值	标准误差	95% 置信区间	
			下限	上限
1.00	3.420	.077	3.269	3.572
2.00	4.145	.077	3.994	4.296

成对比较 ➔ Pairwise Comparisons

因变量: score

(I) typeplc2	(J) typeplc2	平均值差值	标准误差	显著性[b]	差值的 95% 置信区间[b]

		(I-J)			下限	上限
1.00	2.00	-.724*	.109	.000	-.939	-.510
2.00	1.00	.724*	.109	.000	.510	.939

基于估算边际平均值

*. 平均值差值的显著性水平为 .05。

b. 多重比较调节：最低显著差异法（相当于不进行调整）。

单变量检验 ➡ Univariate Tests

因变量: score

	平方和	自由度	均方	F	显著性	偏 Eta 平方
对比	29.753	1	29.753	44.351	.000	.168
误差	146.920	219	.671			

F 检验 typeplc2 的效应。此检验基于估算边际平均值之间的线性无关成对比较。

4. framepln2 * psychologyhlt2

因变量: score

				95% 置信区间	
framepln2	psychologyhlt2	平均值	标准误差	下限	上限
1.00	1.00	3.868	.109	3.654	4.082
	2.00	4.019	.110	3.801	4.237
2.00	1.00	3.501	.109	3.285	3.716
	2.00	3.743	.107	3.533	3.954

5. framepln2 * typeplc2

因变量: score

				95% 置信区间	
framepln2	typeplc2	平均值	标准误差	下限	上限
1.00	1.00	3.714	.110	3.498	3.930
	2.00	4.173	.109	3.957	4.388
2.00	1.00	3.127	.109	2.913	3.341
	2.00	4.117	.108	3.905	4.329

6. psychologyhlt2 * typeplc2

因变量:　score

psychologyhlt2	typeplc2	平均值	标准误差	95% 置信区间 下限	95% 置信区间 上限
1.00	1.00	3.339	.109	3.125	3.552
	2.00	4.030	.109	3.814	4.245
2.00	1.00	3.502	.110	3.287	3.718
	2.00	4.260	.108	4.048	4.472

7. framepln2 * psychologyhlt2 * typeplc2

因变量:　score

framepln2	psychologyhlt2	typeplc2	平均值	标准误差	95% 置信区间 下限	95% 置信区间 上限
1.00	1.00	1.00	3.700	.152	3.400	4.000
		2.00	4.036	.155	3.731	4.341
	2.00	1.00	3.728	.158	3.418	4.039
		2.00	4.310	.155	4.004	4.615
2.00	1.00	1.00	2.977	.155	2.672	3.282
		2.00	4.024	.155	3.719	4.329
	2.00	1.00	3.276	.152	2.977	3.576
		2.00	4.211	.150	3.916	4.505

轮廓图 ➔ Profile Plots

framepln2 * psychologyhlt2 * typeplc2

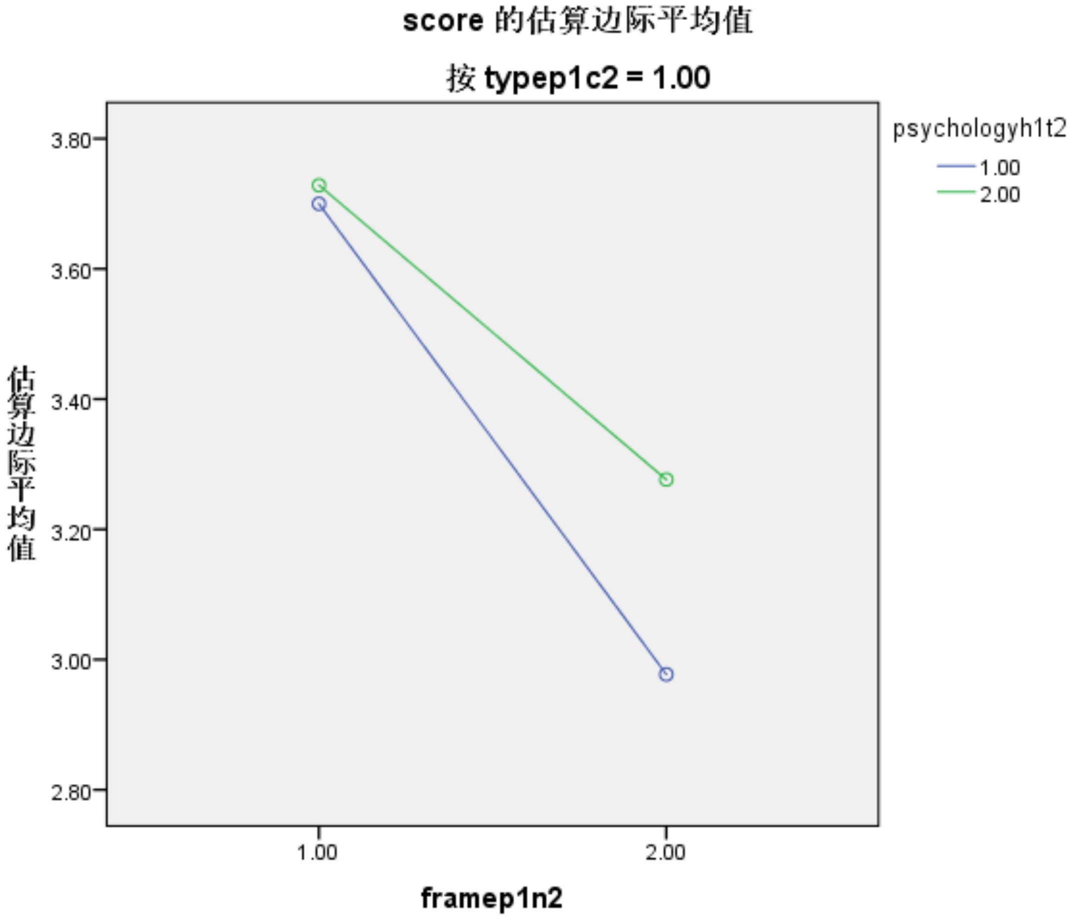
score 的估算边际平均值
按 typep1c2 = 1.00
psychologyh1t2
1.00
2.00
估算边际平均值
3.80
3.60
3.40
3.20
3.00
2.80
1.00
2.00
framep1n2

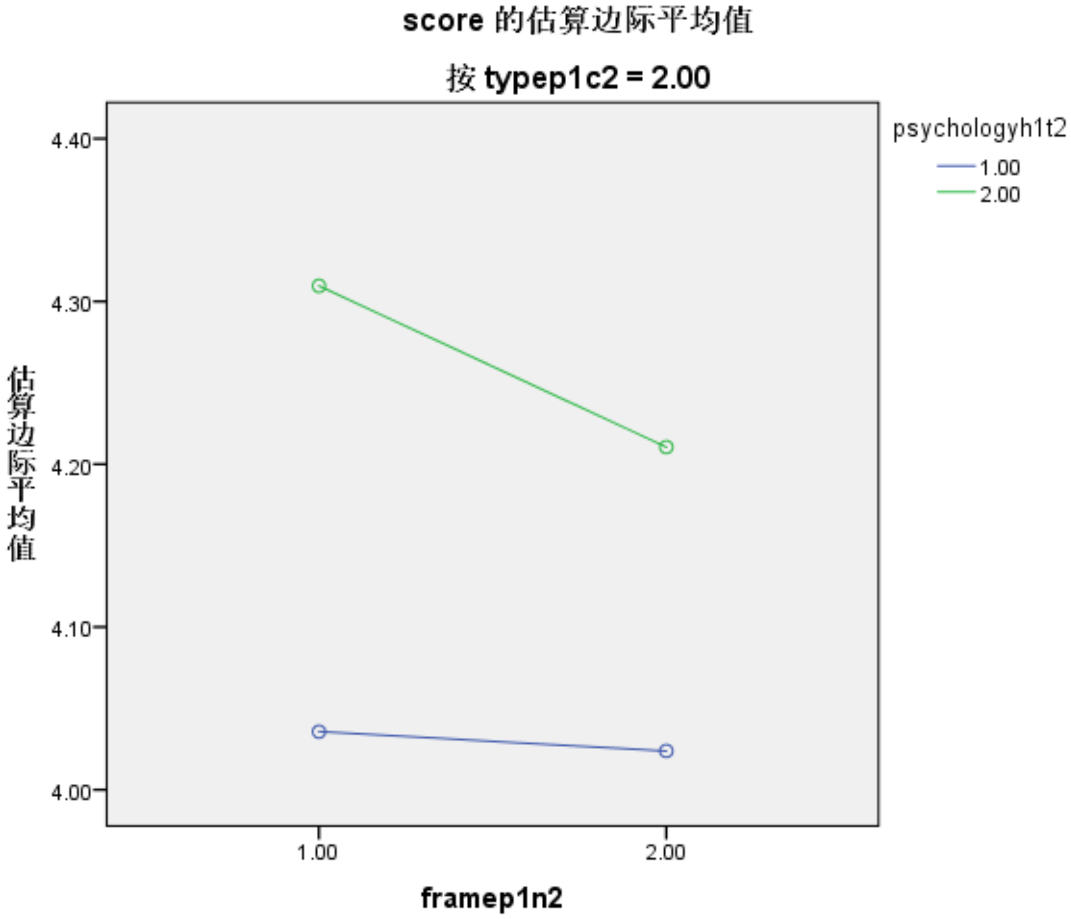

score 的估算边际平均值
按 typep1c2 = 2.00
估算边际平均值
4.40
4.30
4.20
4.10
4.00
1.00
2.00
framep1n2
psychologyh1t2
1.00
2.00

Self-efficacy ANOVA

```
UNIANOVA score BY framep1n2 psychologyh1t2 typep1c2
  /METHOD=SSTYPE(3)
  /INTERCEPT=INCLUDE
  /PLOT=PROFILE(framep1n2*psychologyh1t2*typep1c2)
  /EMMEANS=TABLES(framep1n2) COMPARE ADJ(LSD)
  /EMMEANS=TABLES(psychologyh1t2) COMPARE ADJ(LSD)
  /EMMEANS=TABLES(typep1c2) COMPARE ADJ(LSD)
  /EMMEANS=TABLES(framep1n2*psychologyh1t2)
  /EMMEANS=TABLES(framep1n2*typep1c2)
  /EMMEANS=TABLES(psychologyh1t2*typep1c2)
  /EMMEANS=TABLES(framep1n2*psychologyh1t2*typep1c2)
  /EMMEANS=TABLES(framep1n2*psychologyh1t2) COMPARE(framep1n2)ADJ(SIDAK)
  /EMMEANS=TABLES(framep1n2*psychologyh1t2) COMPARE(psychologyh1t2)ADJ(SIDAK)
 /EMMEANS=TABLES(framep1n2*typep1c2) COMPARE(framep1n2)ADJ(SIDAK)
 /EMMEANS=TABLES(framep1n2*typep1c2) COMPARE(typep1c2)ADJ(SIDAK)
 /EMMEANS=TABLES(psychologyh1t2*typep1c2) COMPARE(psychologyh1t2)ADJ(SIDAK)
 /EMMEANS=TABLES(psychologyh1t2*typep1c2) COMPARE(typep1c2)ADJ(SIDAK)
  /PRINT=ETASQ DESCRIPTIVE
  /CRITERIA=ALPHA(.05)
  /DESIGN=framep1n2 psychologyh1t2 typep1c2 framep1n2*psychologyh1t2 framep1n2*typep1c2
    psychologyh1t2*typep1c2 framep1n2*psychologyh1t2*typep1c2.
```

方差的单变量分析 ➡ One-Way ANOVA

备注

已创建输出		28-APR-2024 16:01:24
注释		
输入	活动数据集	数据集0
	过滤器	<无>
	权重	<无>
	拆分文件	<无>
	工作数据文件中的行数	227
缺失值处理	对缺失的定义	将用户定义的缺失值视为缺失。
	使用的个案数	统计基于所有那些对于模型中的所有变量都具有有效数据的个案。

语法	UNIANOVA score BY framep1n2 psychologyh1t2 typep1c2<br>/METHOD=SSTYPE(3)<br>/INTERCEPT=INCLUDE<br><br>/PLOT=PROFILE(framep1n2*psychologyh1t2*typep1c2)<br>/EMMEANS=TABLES(framep1n2) COMPARE ADJ(LSD)<br>/EMMEANS=TABLES(psychologyh1t2) COMPARE ADJ(LSD)<br>/EMMEANS=TABLES(typep1c2) COMPARE ADJ(LSD)<br><br>/EMMEANS=TABLES(framep1n2*psychologyh1t2)<br><br>/EMMEANS=TABLES(framep1n2*typep1c2)<br><br>/EMMEANS=TABLES(psychologyh1t2*typep1c2)<br><br>/EMMEANS=TABLES(framep1n2*psychologyh1t2*typep1c2)<br><br>/EMMEANS=TABLES(framep1n2*psychologyh1t2) COMPARE(framep1n2)ADJ(SIDAK)<br><br>/EMMEANS=TABLES(framep1n2*psychologyh1t2) COMPARE(psychologyh1t2)ADJ(SIDAK)<br><br>/EMMEANS=TABLES(framep1n2*typep1c2) COMPARE(framep1n2)ADJ(SIDAK)<br><br>/EMMEANS=TABLES(framep1n2*typep1c2) COMPARE(typep1c2)ADJ(SIDAK)<br><br>/EMMEANS=TABLES(psychologyh1t2*typep1c2) COMPARE(psychologyh1t2)ADJ(SIDAK)<br><br>/EMMEANS=TABLES(psychologyh1t2*typep1c2) COMPARE(typep1c2)ADJ(SIDAK)<br>/PRINT=ETASQ DESCRIPTIVE<br>/CRITERIA=ALPHA(.05)<br>/DESIGN=framep1n2 psychologyh1t2 typep1c2 framep1n2*psychologyh1t2

资源	处理程序时间	00:00:00.95
	耗用时间	00:00:00.32

主体间因子 ➡ Between-Subjects Factors

		个案数
framepln2	1.00	112
	2.00	115
psychologyhlt2	1.00	113
	2.00	114
typeplc2	1.00	113
	2.00	114

描述统计 ➡ Descriptive Statistics

因变量: score

framepln2	psychologyhlt2	typeplc2	平均值	标准偏差	个案数
1.00	1.00	1.00	3.6782	.91929	29
		2.00	3.9989	.54569	28
		总计	3.8357	.76961	57
	2.00	1.00	3.9123	.54488	27
		2.00	4.4286	.63413	28
		总计	4.1752	.64175	55
	总计	1.00	3.7911	.76454	56
		2.00	4.2137	.62496	56
		总计	4.0024	.72679	112
2.00	1.00	1.00	4.2733	.53799	28
		2.00	3.8440	.56391	28
		总计	4.0587	.58745	56
	2.00	1.00	3.7802	.55232	29
		2.00	4.1437	.55262	30
		总计	3.9650	.57754	59
	总计	1.00	4.0225	.59492	57
		2.00	3.9990	.57342	58
		总计	4.0106	.58173	115
总计	1.00	1.00	3.9705	.80760	57
		2.00	3.9215	.55533	56
		总计	3.9462	.69149	113
	2.00	1.00	3.8439	.54781	56
		2.00	4.2812	.60537	58

		总计	4.0664	.61574	114
	总计	1.00	3.9078	.69102	113
		2.00	4.1045	.60629	114
		总计	4.0066	.65586	227

主体间效应检验 ➡ Tests of Between-Subjects Effects

因变量: score

源	III 类平方和	自由度	均方	F	显著性	偏 Eta 平方
修正模型	13.137[a]	7	1.877	4.888	.000	.135
截距	3642.180	1	3642.180	9486.864	.000	.977
framepln2	.002	1	.002	.005	.944	.000
psychologyhlt2	.784	1	.784	2.042	.154	.009
typeplc2	2.107	1	2.107	5.489	.020	.024
framepln2 * psychologyhlt2	2.605	1	2.605	6.784	.010	.030
framepln2 * typeplc2	2.889	1	2.889	7.524	.007	.033
psychologyhlt2 * typeplc2	3.460	1	3.460	9.013	.003	.040
framepln2 * psychologyhlt2 * typeplc2	1.264	1	1.264	3.293	.071	.015
误差	84.078	219	.384			
总计	3741.172	227				
修正后总计	97.215	226				

a. R 方 = .135（调整后 R 方 = .107）

估算边际平均值 ➡ Estimated Marginal Means

1. framepln2

估算值 ➡ Estimates

因变量: score

framepln2	平均值	标准误差	95% 置信区间	
			下限	上限
1.00	4.005	.059	3.889	4.120
2.00	4.010	.058	3.896	4.124

成对比较 ➡ Pairwise Comparisons

因变量: score

(I) framepln2	(J) framepln2	平均值差值 (I-J)	标准误差	显著性[a]	差值的 95% 置信区间[a]	
					下限	上限
1.00	2.00	-.006	.082	.944	-.168	.156
2.00	1.00	.006	.082	.944	-.156	.168

基于估算边际平均值

a．多重比较调节：最低显著差异法（相当于不进行调整）。

单变量检验 ➡ Univariate Tests

因变量:　score

	平方和	自由度	均方	F	显著性	偏 Eta 平方
对比	.002	1	.002	.005	.944	.000
误差	84.078	219	.384			

F 检验 framep1n2 的效应。此检验基于估算边际平均值之间的线性无关成对比较。

2. psychologyh1t2

估算值 ➡ Estimates

因变量:　score

psychologyh1t2	平均值	标准误差	95% 置信区间	
			下限	上限
1.00	3.949	.058	3.834	4.064
2.00	4.066	.058	3.952	4.181

成对比较 ➡ Pairwise Comparisons

因变量:　score

(I) psychologyh1t2	(J) psychologyh1t2	平均值差值 (I-J)	标准误差	显著性[a]	差值的 95% 置信区间[a]	
					下限	
1.00	2.00	-.118	.082	.154	-.280	
2.00	1.00	.118	.082	.154	-.045	

成对比较 ➡ Pairwise Comparisons

因变量:　score

(I) psychologyh1t2	(J) psychologyh1t2	差值的 95% 置信区间
		上限
1.00	2.00	.045
2.00	1.00	.280

基于估算边际平均值

a．多重比较调节：最低显著差异法（相当于不进行调整）。

单变量检验 ➡ Univariate Tests

因变量:　score

	平方和	自由度	均方	F	显著性	偏 Eta 平方
对比	.784	1	.784	2.042	.154	.009
误差	84.078	219	.384			

F 检验 psychologyh1t2 的效应。此检验基于估算边际平均值之间的线性无关成对比较。

3. typep1c2

估算值 ➡ Estimates

因变量: score

typep1c2	平均值	标准误差	95% 置信区间	
			下限	上限
1.00	3.911	.058	3.796	4.026
2.00	4.104	.058	3.989	4.218

成对比较 ➡ Pairwise Comparisons

因变量: score

(I) typep1c2	(J) typep1c2	平均值差值 (I-J)	标准误差	显著性[b]	差值的 95% 置信区间[b]	
					下限	上限
1.00	2.00	-.193*	.082	.020	-.355	-.031
2.00	1.00	.193*	.082	.020	.031	.355

基于估算边际平均值

*. 平均值差值的显著性水平为 .05。

b. 多重比较调节：最低显著差异法（相当于不进行调整）。

单变量检验 ➡ Univariate Tests

因变量: score

	平方和	自由度	均方	F	显著性	偏 Eta 平方
对比	2.107	1	2.107	5.489	.020	.024
误差	84.078	219	.384			

F 检验 typep1c2 的效应。此检验基于估算边际平均值之间的线性无关成对比较。

4. framep1n2 * psychologyh1t2

因变量: score

framep1n2	psychologyh1t2	平均值	标准误差	95% 置信区间	
				下限	上限
1.00	1.00	3.839	.082	3.677	4.000
	2.00	4.170	.084	4.006	4.335
2.00	1.00	4.059	.083	3.896	4.222
	2.00	3.962	.081	3.803	4.121

5. framep1n2 * typep1c2

因变量: score

framep1n2	typep1c2	平均值	标准误差	95% 置信区间	
				下限	上限
1.00	1.00	3.795	.083	3.632	3.959
	2.00	4.214	.083	4.051	4.377
2.00	1.00	4.027	.082	3.865	4.189

2.00	3.994	.081	3.833	4.154

6. psychologyh1t2 * typep1c2

因变量: score

psychologyh1t2	typep1c2	平均值	标准误差	95% 置信区间 下限	95% 置信区间 上限
1.00	1.00	3.976	.082	3.814	4.138
	2.00	3.921	.083	3.758	4.085
2.00	1.00	3.846	.083	3.683	4.010
	2.00	4.286	.081	4.126	4.447

7. framep1n2 * psychologyh1t2 * typep1c2

因变量: score

framep1n2	psychologyh1t2	typep1c2	平均值	标准误差	95% 置信区间 下限	95% 置信区间 上限
1.00	1.00	1.00	3.678	.115	3.451	3.905
		2.00	3.999	.117	3.768	4.230
	2.00	1.00	3.912	.119	3.677	4.147
		2.00	4.429	.117	4.198	4.659
2.00	1.00	1.00	4.273	.117	4.043	4.504
		2.00	3.844	.117	3.613	4.075
	2.00	1.00	3.780	.115	3.553	4.007
		2.00	4.144	.113	3.921	4.367

8. framep1n2 * psychologyh1t2

估算值 ➜ Estimates

因变量: score

framep1n2	psychologyh1t2	平均值	标准误差	95% 置信区间 下限	95% 置信区间 上限
1.00	1.00	3.839	.082	3.677	4.000
	2.00	4.170	.084	4.006	4.335
2.00	1.00	4.059	.083	3.896	4.222
	2.00	3.962	.081	3.803	4.121

成对比较 ➜ Pairwise Comparisons

因变量: score

psychologyh1t2	(I) framep1n2	(J) framep1n2	平均值差值 (I-J)	标准误差	显著性[a]	差值的 95% 置信区间[a] 下限	

1.00	1.00	2.00	-.220	.117	.060	-.450	
	2.00	1.00	.220	.117	.060	-.010	
2.00	1.00	2.00	.209	.116	.074	-.020	
	2.00	1.00	-.209	.116	.074	-.437	

成对比较 → Pairwise Comparisons

因变量:　score

psychologyh1t2	(I) framepln2	(J) framepln2	差值的 95% 置信区间 上限
1.00	1.00	2.00	.010
	2.00	1.00	.450
2.00	1.00	2.00	.437
	2.00	1.00	.020

基于估算边际平均值

a. 多重比较调节：斯达克法。

单变量检验 → Univariate Tests

因变量:　score

psychologyh1t2		平方和	自由度	均方	F	显著性	偏 Eta 平方
1.00	对比	1.369	1	1.369	3.565	.060	.016
	误差	84.078	219	.384			
2.00	对比	1.237	1	1.237	3.222	.074	.015
	误差	84.078	219	.384			

每个 F 都将检验其他所示效应的每个级别组合中 framepln2 的简单效应。这些检验基于估算边际平均值之间的线性无关成对比较。

9. framepln2 * psychologyh1t2

估算值 → Estimates

因变量:　score

framepln2	psychologyh1t2	平均值	标准误差	95% 置信区间 下限	95% 置信区间 上限
1.00	1.00	3.839	.082	3.677	4.000
	2.00	4.170	.084	4.006	4.335
2.00	1.00	4.059	.083	3.896	4.222
	2.00	3.962	.081	3.803	4.121

成对比较 → Pairwise Comparisons

因变量:　score

framepln2	(I) psychologyhlt2	(J) psychologyhlt2	平均值差值 (I-J)	标准误差	显著性[b]		
1.00	1.00	2.00	-.332*	.117	.005		
	2.00	1.00	.332*	.117	.005		
2.00	1.00	2.00	.097	.116	.404		
	2.00	1.00	-.097	.116	.404		

成对比较 → Pairwise Comparisons

因变量:　score

framepln2	(I) psychologyhlt2	(J) psychologyhlt2	差值的 95% 置信区间[b]	
			下限	上限
1.00	1.00	2.00	-.563	-.101
	2.00	1.00	.101	.563
2.00	1.00	2.00	-.131	.325
	2.00	1.00	-.325	.131

基于估算边际平均值

*. 平均值差值的显著性水平为 .05。

b. 多重比较调节：斯达克法。

单变量检验 → Univariate Tests

因变量:　score

framepln2		平方和	自由度	均方	F	显著性	偏 Eta 平方
1.00	对比	3.083	1	3.083	8.030	.005	.035
	误差	84.078	219	.384			
2.00	对比	.269	1	.269	.700	.404	.003
	误差	84.078	219	.384			

每个 F 都将检验其他所示效应的每个级别组合中 psychologyhlt2 的简单效应。这些检验基于估算边际平均值之间的线性无关成对比较。

10. framepln2 * typeplc2

估算值 → Estimates

因变量:　score

framepln2	typeplc2	平均值	标准误差	95% 置信区间	
				下限	上限
1.00	1.00	3.795	.083	3.632	3.959
	2.00	4.214	.083	4.051	4.377
2.00	1.00	4.027	.082	3.865	4.189

	2.00	3.994	.081	3.833	4.154

成对比较 ➡ Pairwise Comparisons

因变量:　score

typeplc2	(I) framepln2	(J) framepln2	平均值差值 (I-J)	标准误差	显著性[b]	差值的 95% 置信区间[b] 下限
1.00	1.00	2.00	-.232[*]	.117	.048	-.461
	2.00	1.00	.232[*]	.117	.048	.002
2.00	1.00	2.00	.220	.116	.060	-.009
	2.00	1.00	-.220	.116	.060	-.449

成对比较 ➡ Pairwise Comparisons

因变量:　score

typeplc2	(I) framepln2	(J) framepln2	差值的 95% 置信区间 上限
1.00	1.00	2.00	-.002
	2.00	1.00	.461
2.00	1.00	2.00	.449
	2.00	1.00	.009

基于估算边际平均值

*. 平均值差值的显著性水平为 .05。

b. 多重比较调节：斯达克法。

单变量检验 ➡ Univariate Tests

因变量:　score

typeplc2		平方和	自由度	均方	F	显著性	偏 Eta 平方
1.00	对比	1.513	1	1.513	3.941	.048	.018
	误差	84.078	219	.384			
2.00	对比	1.377	1	1.377	3.586	.060	.016
	误差	84.078	219	.384			

每个 F 都将检验其他所示效应的每个级别组合中 framepln2 的简单效应。这些检验基于估算边际平均值之间的线性无关成对比较。

11. framepln2 * typeplc2

估算值 ➡ Estimates

因变量:　score

framepln2	typeplc2	平均值	标准误差	95% 置信区间 下限	95% 置信区间 上限
1.00	1.00	3.795	.083	3.632	3.959
	2.00	4.214	.083	4.051	4.377

2.00	1.00	4.027	.082	3.865	4.189
	2.00	3.994	.081	3.833	4.154

成对比较 ➔ Pairwise Comparisons

因变量: score

frameln2	(I) typeplc2	(J) typeplc2	平均值差值 (I-J)	标准误差	显著性[b]	差值的 95% 置信区间[b]	
						下限	
1.00	1.00	2.00	-.418[*]	.117	.000	-.649	
	2.00	1.00	.418[*]	.117	.000	.188	
2.00	1.00	2.00	.033	.116	.776	-.195	
	2.00	1.00	-.033	.116	.776	-.261	

成对比较 ➔ Pairwise Comparisons

因变量: score

frameln2	(I) typeplc2	(J) typeplc2	差值的 95% 置信区间
			上限
1.00	1.00	2.00	-.188
	2.00	1.00	.649
2.00	1.00	2.00	.261
	2.00	1.00	.195

基于估算边际平均值

*. 平均值差值的显著性水平为 .05。

b. 多重比较调节：斯达克法。

单变量检验 ➔ Univariate Tests

因变量: score

frameln2		平方和	自由度	均方	F	显著性	偏 Eta 平方
1.00	对比	4.901	1	4.901	12.765	.000	.055
	误差	84.078	219	.384			
2.00	对比	.031	1	.031	.081	.776	.000
	误差	84.078	219	.384			

每个 F 都将检验其他所示效应的每个级别组合中 typeplc2 的简单效应。这些检验基于估算边际平均值之间的线性无关成对比较。

12. psychologyhlt2 * typeplc2

估算值 ➔ Estimates

因变量: score

psychologyhlt2	typeplc2	平均值	标准误差	95% 置信区间	
				下限	上限

1.00	1.00	3.976	.082	3.814	4.138
	2.00	3.921	.083	3.758	4.085
2.00	1.00	3.846	.083	3.683	4.010
	2.00	4.286	.081	4.126	4.447

成对比较 ➔ Pairwise Comparisons

因变量: score

typeplc 2	(I) psychologyh1t2	(J) psychologyh1t2	平均值差值 (I-J)	标准误差	显著性[b]		
1.00	1.00	2.00	.129	.117	.268		
	2.00	1.00	-.129	.117	.268		
2.00	1.00	2.00	-.365*	.116	.002		
	2.00	1.00	.365*	.116	.002		

成对比较 ➔ Pairwise Comparisons

因变量: score

typeplc2	(I) psychologyh1t2	(J) psychologyh1t2	差值的 95% 置信区间[b]	
			下限	上限
1.00	1.00	2.00	-.100	.359
	2.00	1.00	-.359	.100
2.00	1.00	2.00	-.593	-.136
	2.00	1.00	.136	.593

基于估算边际平均值

*. 平均值差值的显著性水平为 .05。

b. 多重比较调节：斯达克法。

单变量检验 ➔ Univariate Tests

因变量: score

typeplc2		平方和	自由度	均方	F	显著性	偏 Eta 平方
1.00	对比	.473	1	.473	1.232	.268	.006
	误差	84.078	219	.384			
2.00	对比	3.786	1	3.786	9.861	.002	.043
	误差	84.078	219	.384			

每个 F 都将检验其他所示效应的每个级别组合中 psychologyh1t2 的简单效应。这些检验基于估算边际平均值之间的线性无关成对比较。

13. psychologyh1t2 * typeplc2

估算值 ➔ Estimates

因变量: score

psychologyh1t2	typeplc2	平均值	标准误差	95% 置信区间	
				下限	上限
1.00	1.00	3.976	.082	3.814	4.138

	2.00	3.921	.083	3.758	4.085
2.00	1.00	3.846	.083	3.683	4.010
	2.00	4.286	.081	4.126	4.447

成对比较 ➜ Pairwise Comparisons

因变量:　score

psychologyhlt2	(I) typeplc2	(J) typeplc2	平均值差值 (I-J)	标准误差	显著性[b]	差值的 95% 置信区间[b]	
						下限	
1.00	1.00	2.00	.054	.117	.642	-.176	
	2.00	1.00	-.054	.117	.642	-.284	
2.00	1.00	2.00	-.440*	.116	.000	-.669	
	2.00	1.00	.440*	.116	.000	.211	

成对比较 ➜ Pairwise Comparisons

因变量:　score

psychologyhlt2	(I) typeplc2	(J) typeplc2	差值的 95% 置信区间
			上限
1.00	1.00	2.00	.284
	2.00	1.00	.176
2.00	1.00	2.00	-.211
	2.00	1.00	.669

基于估算边际平均值

*. 平均值差值的显著性水平为 .05。

b. 多重比较调节：斯达克法。

单变量检验 ➜ Univariate Tests

因变量:　score

psychologyhlt2		平方和	自由度	均方	F	显著性	偏 Eta 平方
1.00	对比	.083	1	.083	.217	.642	.001
	误差	84.078	219	.384			
2.00	对比	5.505	1	5.505	14.339	.000	.061
	误差	84.078	219	.384			

每个 F 都将检验其他所示效应的每个级别组合中 typeplc2 的简单效应。这些检验基于估算边际平均值之间的线性无关成对比较。
